# Distinct trajectory patterns of neutrophil-to-albumin ratio predict clinical outcomes after endovascular therapy in large vessel occlusion stroke

**DOI:** 10.3389/fnagi.2025.1570662

**Published:** 2025-06-04

**Authors:** Weiwei Gao, Junxuan Sun, Lingfeng Yu, Jingjing She, Yanan Zhao, Lijuan Cai, Xingyu Chen, Renjing Zhu

**Affiliations:** ^1^Department of Neurology, Zhongshan Hospital of Xiamen University, School of Medicine, Xiamen University, Xiamen, China; ^2^Department of Emergency, Zhongshan Hospital of Xiamen University, School of Medicine, Xiamen University, Xiamen, China; ^3^School of Medicine, Xiamen University, Xiamen, China; ^4^The School of Clinical Medicine, Fujian Medical University, Fuzhou, Fujian, China; ^5^Xiamen Clinical Research Center for Cerebrovascular Diseases, Xiamen, China

**Keywords:** acute ischemic stroke, large vessel occlusion stroke, endovascular therapy, neutrophil-to-albumin ratio, clinical outcomes, latent class trajectory modeling, propensity score matching

## Abstract

**Objective:**

To investigate the dynamic characteristics and prognostic value of neutrophil-to-albumin ratio (NAR) in patients with acute large vessel occlusion ischemic stroke (LVO-AIS) undergoing endovascular therapy (EVT).

**Methods:**

In this retrospective cohort study, we consecutively enrolled 299 patients with anterior circulation LVO-AIS who underwent EVT between January 2018 and February 2024. NAR was measured at admission, day 1, and day 3 after EVT. The primary outcome was poor functional outcome at 90 days (modified Rankin Scale score 3–6). Secondary outcomes included symptomatic intracranial hemorrhage (sICH), malignant cerebral edema (MCE), and in-hospital mortality (IHM). Multivariable logistic regression and restricted cubic spline regression were used to analyze the association between NAR and functional outcomes. Latent class trajectory modeling (LCTM) was applied to identify NAR evolution patterns, and propensity score matching (PSM) was performed to balance baseline characteristics between different trajectory groups, followed by conditional logistic regression to assess their association with clinical outcomes.

**Results:**

At 90-day follow-up, 197 patients (65.9%) had poor outcomes. The predictive value of NAR increased over time, with day 3 NAR showing the best predictive performance (poor outcome: AUC = 0.79; sICH: AUC = 0.70; MCE: AUC = 0.75; IHM: AUC = 0.81). Multivariable analysis showed that for each unit increase in day 3 NAR, the risk of 90-day poor outcome increased 2.81-fold (95% CI: 1.96–4.03, *p* < 0.001). LCTM analysis identified two distinct NAR evolution patterns: continuously increasing (31.1%) and peak-then-decline (68.7%). After PSM (63 patients per group), compared with the continuously increasing trajectory, the peak-then-decline trajectory was associated with significantly lower risks of poor functional outcome (OR = 0.38, 95% CI: 0.17–0.86, *p* = 0.020), sICH (OR = 0.38, 95% CI: 0.17–0.86, p = 0.020), MCE (OR = 0.25, 95% CI: 0.10–0.61, *p* = 0.002), and IHM (OR = 0.13, 95% CI: 0.04–0.42, *p* < 0.001).

**Conclusion:**

NAR trajectory patterns independently predict clinical outcomes after EVT for LVO-AIS. Dynamic monitoring of NAR, particularly on day 3 post-procedure, may facilitate early risk stratification and development of targeted intervention strategies, providing a new biomarker tool for precision stroke management.

## 1 Introduction

Acute ischemic stroke (AIS) remains one of the leading causes of mortality and disability worldwide, with large vessel occlusion (LVO) strokes accounting for 28–46% of all AIS cases (Feigin et al., 2025; Smith et al., 2009). These patients warrant particular clinical attention due to their rapid disease progression, severe neurological deficits, and poor prognosis (Malhotra et al., 2017). Endovascular therapy (EVT), by rapidly recanalizing occluded vessels and salvaging the ischemic penumbra, has been proven to significantly improve patient outcomes and has become the standard treatment strategy for acute large vessel occlusion ischemic stroke (LVO-AIS) in the hyperacute phase (Dhillon et al., 2022). However, clinical practice reveals that despite successful vessel recanalization, a considerable proportion of patients still experience poor outcomes. Additionally, nearly half of patients develop postoperative complications such as hemorrhagic transformation (HT) and cerebral edema, significantly increasing the risk of early mortality and long-term functional disability (Hao et al., 2017; Kimberly et al., 2018). Therefore, early identification of reliable prognostic biomarkers after EVT to achieve precise risk stratification remains a critical unresolved issue in current stroke management.

Neuroinflammation plays a pivotal role in the ischemia–reperfusion injury cascade. Neutrophils, as the primary effector cells in the inflammatory response, participate in multiple stages of disease progression through the release of reactive oxygen species, metalloproteinases, and neurotoxic mediators, including disruption of blood–brain barrier (BBB) integrity, exacerbation of neuronal damage, and inhibition of neuroplasticity (Kang et al., 2020). Conversely, albumin serves not only as a sensitive indicator of nutritional status but also maintains vascular endothelial function integrity through multiple mechanisms including antioxidant, anti-inflammatory, and anti-thrombotic effects, thereby protecting the BBB from inflammation-mediated damage (Don and Kaysen, 2004). The neutrophil-to-albumin ratio (NAR), as a composite indicator integrating both inflammatory intensity and nutritional status, has recently demonstrated excellent prognostic value in various pathological conditions, including malignant tumors, acute myocardial infarction, heart failure, hemorrhagic stroke, and autoimmune diseases (Deng et al., 2024; Hu et al., 2022; Kamal et al., 2023; Karasu et al., 2023; Xie et al., 2024). In the field of ischemic stroke, although studies have preliminarily revealed significant associations between NAR and adverse outcomes, these explorations have primarily been limited to single time-point NAR measurements, failing to fully capture the dynamic balance of inflammatory-nutritional status and its clinical significance (Bao et al., 2024; Zhao et al., 2023).

Growing evidence suggests that the dynamic pattern of inflammatory marker changes provides a more comprehensive and accurate reflection of disease progression and clinical outcomes in AIS patients compared to single time-point measurements. Previous studies have found that inflammatory markers measured on day 1 or day 3 demonstrate stronger predictive capability for key clinical outcomes in AIS compared to baseline values (Wu and Chen, 2023; Guo et al., 2016; Zhang et al., 2024; Ying et al., 2020; Qian et al., 2024; Weng et al., 2021). However, several important knowledge gaps remain in current research. First, existing studies have mainly focused on general AIS patient populations, with very limited research on LVO-AIS patients who have larger infarct volumes and worse prognoses. Second, no studies have investigated the trajectory patterns of inflammatory markers and their predictive value for clinical outcomes.

Based on this research background, the present study aims to explore the dynamic characteristics and prognostic value of NAR in anterior circulation LVO-AIS patients undergoing EVT. By quantitatively analyzing NAR changes at different time points and their trajectory patterns, we seek to identify high-risk patient subgroups and evaluate their associations with major clinical outcomes. We hypothesize that different NAR trajectory patterns may represent distinct regulatory modes of pathophysiological response, and the differentiation of these patterns could provide new theoretical foundations for early risk stratification, precision monitoring, and potential targeted anti-inflammatory intervention strategies in stroke patients.

## 2 Materials and methods

### 2.1 Study design and population

We conducted a retrospective cohort study using a prospectively maintained database. The study protocol was approved by the institutional review board, and the requirement for informed consent was waived due to the retrospective nature of the study. We consecutively enrolled adult patients (aged ≥18 years) with anterior circulation LVO-AIS who underwent EVT between January 2018 and February 2024. All enrolled patients had confirmed anterior circulation LVO prior to EVT, documented by computed tomography angiography, magnetic resonance angiography, or digital subtraction angiography (DSA) during the procedure. LVO was defined as occlusion of the internal carotid artery (ICA) or M1/M2 segments of the middle cerebral artery. Exclusion criteria included: (1) pre-stroke modified Rankin Scale (mRS) score >2; (2) concomitant severe systemic diseases such as renal failure, severe hepatic insufficiency, or malignancy; (3) missing critical clinical data; and (4) loss to 90-day follow-up.

### 2.2 Data collection and clinical assessment

Baseline characteristics included demographic features (age and sex), cerebrovascular risk factors (current smoker, alcohol consumption, hypertension, diabetes mellitus, hyperlipidemia, atrial fibrillation, previous stroke or transient ischemic attack, and coronary artery disease), and vital signs at admission (systolic and diastolic blood pressure). Stroke severity was assessed using the National Institutes of Health Stroke Scale (NIHSS) at admission, and the level of consciousness was evaluated using the Glasgow Coma Scale (GCS). The Alberta Stroke Program Early CT Score (ASPECTS) was used to quantify baseline infarct volume. Stroke etiology was classified according to the Trial of Org 10,172 in Acute Stroke Treatment criteria.

Procedure-related parameters were recorded in real time by the operators, including intravenous thrombolysis status, vessel occlusion details (ICA, M1 segment of middle cerebral artery, M2 segment of middle cerebral artery, or tandem lesions), key time points (onset-to-puncture time, onset-to-reperfusion time, and puncture-to-recanalization time [PRT]), and procedural details (number of mechanical thrombectomy attempts, device strategy [stent retriever, aspiration catheter, or combined], and balloon angioplasty). Tandem lesions were defined as severe stenosis (70–99%) or occlusion of the ICA with concomitant ipsilateral occlusion of the ICA terminus, middle cerebral artery, or anterior cerebral artery. Reperfusion status was assessed using the modified Thrombolysis in Cerebral Ischemia (mTICI) scoring system based on final DSA results, with mTICI grades 2b-3 defined as successful reperfusion.

### 2.3 Laboratory measurements

Venous blood samples were collected in the fasting state at admission (baseline), day 1, and day 3 after EVT. All assays were performed by the hospital’s central laboratory using standardized procedures. Laboratory parameters included complete blood count (white blood cells, neutrophils, lymphocytes, red blood cells, hemoglobin, and platelets), biochemical indicators (total protein, albumin, creatinine, and uric acid), and lipid profile (total cholesterol, triglycerides, high-density lipoprotein cholesterol, and low-density lipoprotein cholesterol). Based on these laboratory parameters, we calculated the NAR at each time point (baseline NAR, day 1 NAR, and day 3 NAR) using the formula: NAR = neutrophil count (×10^9^/L)/albumin concentration (g/dL). Additionally, we calculated the average values of neutrophils, albumin, and NAR across the three time points.

### 2.4 Outcome measures

The primary clinical outcome was poor functional outcome at 90 days, defined as a 90-day mRS score of 3–6. Secondary outcomes included symptomatic intracranial hemorrhage (sICH), malignant cerebral edema (MCE), and in-hospital mortality (IHM). All patients underwent head CT scans immediately after EVT, at 24 h, and at 72 h. Additional CT scans were performed immediately upon neurological deterioration or if previous imaging suggested edema progression. sICH was defined as any intracranial hemorrhage accompanied by one of the following conditions without other explainable causes: an increase in the total NIHSS score by ≥4 points, an increase in any single NIHSS item score by ≥2 points from baseline, or neurological deterioration requiring endotracheal intubation, decompressive craniectomy, external ventricular drainage, or other significant medical interventions. MCE was defined as a midline shift >5 mm at the level of the septum pellucidum or pineal gland, accompanied by compression of the perimesencephalic cistern or the need for decompressive craniectomy (Ong et al., 2017).

Follow-up data were obtained through the National Cerebrovascular Disease Big Data Platform (Stroke Center Construction Information Management System). Trained follow-up personnel completed the 90-day mRS functional outcome assessment via telephone and promptly recorded the results in the system. To ensure data quality, two professionally trained neurologists independently collected and recorded all data according to standardized procedures, followed by cross-checking by other researchers.

### 2.5 Statistical analysis

Statistical analyses were performed using R software (version 4.2.2). Normality of continuous variables was assessed using the Shapiro–Wilk test. Normally distributed continuous variables were presented as mean ± standard deviation (Mean ± SD) and compared using independent samples t-tests, while non-normally distributed continuous variables were presented as median (interquartile range [IQR]) and compared using Mann–Whitney *U* tests. Categorical variables were presented as frequencies (percentages) and compared using chi-square tests or Fisher’s exact tests based on expected frequencies and sample size.

To investigate the association between NAR and clinical outcomes, we constructed three progressively adjusted multivariable logistic regression models. Covariate selection followed these steps: first, variables with statistical significance (*p* < 0.05) in univariate analysis were selected as candidate covariates; second, all candidate covariates underwent multicollinearity diagnostics with calculation of variance inflation factor (VIF) and tolerance, and variables not meeting preset criteria (VIF < 10 and Tolerance>0.1) were excluded (Supplementary Table 1). We established three progressively adjusted models: Model 1 was the unadjusted baseline model; Model 2 adjusted for key clinical factors (age, diabetes, atrial fibrillation, baseline NIHSS score, GCS score, ASPECTS); Model 3 further adjusted for procedure-related parameters and laboratory indices (occlusion vessel type, PRT, number of thrombectomy passes, reperfusion status, and lymphocyte count). To assess potential non-linear associations between NAR and clinical outcomes, we employed restricted cubic spline (RCS) regression with knots set at the 5th, 35th, 65th, and 95th percentiles of the data distribution.

For the dynamic characteristics of NAR, we implemented latent class trajectory modeling (LCTM) analysis for all study subjects, determining the optimal number of trajectory categories by comprehensively comparing Bayesian information criterion values and log-likelihood ratios, ensuring each trajectory group contained at least 5% of the sample (Supplementary Table 2). To reduce selection bias due to imbalanced baseline characteristics across trajectory groups, we employed propensity score matching (PSM). Variables with between-group comparison *p*-values <0.2 were selected as matching variables. We used nearest-neighbor matching algorithm for 1:1 exact matching with a caliper width of 0.1 standard deviation units to maximize retained effective sample size while ensuring matching quality. Matching quality was assessed by comparing pre- and post-matching *p*-values for covariate balance, with *p*-values >0.05 indicating balanced distribution. After matching, conditional logistic regression analysis was used to evaluate the strength of association between different NAR trajectory patterns and clinical outcomes.

Additionally, we assessed the predictive value of NAR and its different trajectory patterns for clinical outcomes using receiver operating characteristic (ROC) curve analysis, calculating the area under the curve (AUC), optimal cutoff values, sensitivity, and specificity. For missing data handling, we excluded variables with missing proportions exceeding 10%; for variables with missing proportions below 10%, we employed single imputation using mean or median values based on data distribution characteristics. All statistical tests were two-sided, with *p* < 0.05 defined as statistically significant.

## 3 Results

### 3.1 Patient characteristics

This study included 299 LVO-AIS patients who underwent EVT (Table 1). The median age was 68 years (IQR, 58–76 years), and 194 (64.9%) were male. At 90-day follow-up, 197 (65.9%) patients had poor outcomes (mRS 3–6). During hospitalization, 129 patients (43.1%) experienced HT, of which 68 (22.7%) had sICH. Additionally, 68 (22.7%) patients developed MCE, and 51 (17.1%) experienced IHM.

**Table 1:** Baseline characteristics of patients according to 90-day functional outcome.

Variables	Overall (*n* = 299)	Good outcome (*n* = 102)	Poor outcome (*n* = 197)	*p*-value
Age, years	68 (58, 76)	64 (55, 72)	70 (59, 78)	**0.002**
Male sex	194 (64.88)	73 (71.57)	121 (61.42)	0.081
Current smoker	104 (34.78)	43 (42.16)	61 (30.96)	0.054
Alcohol consumption	71 (23.75)	26 (25.49)	45 (22.84)	0.610
Medical history				
Hypertension	201 (67.22)	63 (61.76)	138 (70.05)	0.148
Diabetes mellitus	84 (28.09)	21 (20.59)	63 (31.98)	**0.038**
Hyperlipidemia	62 (20.74)	23 (22.55)	39 (19.80)	0.578
Atrial fibrillation	133 (44.48)	35 (34.31)	98 (49.75)	**0.011**
Previous stroke/TIA	48 (16.05)	14 (13.73)	34 (17.26)	0.430
Coronary artery disease	40 (13.38)	12 (11.76)	28 (14.21)	0.555
Clinical presentation				
SBP, mmHg	149 (133, 164)	149 (134, 162)	150 (132, 165)	0.733
DBP, mmHg	87 (77, 97)	86 (75, 97)	87 (78, 97)	0.417
Baseline NIHSS score	15 (12, 19)	13 (8, 15)	16 (13, 20)	**<0.001**
Baseline GCS score	12 (10, 14)	14 (12, 15)	11 (9, 14)	**<0.001**
Baseline ASPECT score	9 (8, 10)	9 (8, 10)	9 (7, 10)	**<0.001**
Stroke etiology				0.180
Large-artery atherosclerosis	138 (46.15)	54 (52.94)	84 (42.64)	
Cardioembolism	148 (49.50)	43 (42.16)	105 (53.30)	
Other	13 (4.35)	5 (4.90)	8 (4.06)	
Occlusion site				0.008
ICA	54 (18.06)	13 (12.75)	41 (20.81)	
MCA M1	144 (48.16)	63 (61.76)	81 (41.12)	
MCA M2	26 (8.70)	8 (7.84)	18 (9.14)	
Tandem lesions	75 (25.08)	18 (17.65)	57 (28.93)	
Procedural characteristics				
Intravenous thrombolysis	129 (43.14)	48 (47.06)	81 (41.12)	0.325
OPT, min	368 (260, 544)	335 (231, 560)	372 (269, 515)	0.574
PRT, min	80 (55, 100)	65 (44, 94)	80 (60, 104)	**<0.001**
ORT, min	455 (330, 636)	418 (286, 666)	460 (351, 618)	0.303
NOTA	2 (1, 3)	1 (1, 2)	2 (1, 3)	**0.047**
Successful reperfusion	255 (85.28)	95 (93.14)	160 (81.22)	**0.006**
Treatment strategy				0.066
Stent retriever	68 (22.74)	26 (25.49)	42 (21.32)	
Aspiration	16 (5.35)	6 (5.88)	10 (5.08)	
Combined approach	197 (65.89)	69 (67.65)	128 (64.97)	
Balloon angioplasty	50 (16.72)	13 (12.75)	37 (18.78)	0.185

Data are presented as median (interquartile range) for continuous variables and number (percentage) for categorical variables. TIA, transient ischemic attack; SBP, systolic blood pressure; DBP, diastolic blood pressure; NIHSS, National Institutes of Health Stroke Scale; GCS, Glasgow Coma Scale; ASPECTS, Alberta Stroke Program Early CT Score; ICA, internal carotid artery; MCA, middle cerebral artery; OPT, onset-to-puncture time; PRT, puncture-to-reperfusion time; ORT, onset-to-reperfusion time; NOTA, number of thrombectomy attempts. Bold values indicate *p* < 0.05, representing statistically significant results.

Compared to the good outcome group, patients in the poor outcome group were older (*p* = 0.002) and had higher prevalence of diabetes mellitus (*p* = 0.038) and atrial fibrillation (*p* = 0.011). The poor outcome group had more severe baseline neurological deficits, as indicated by higher NIHSS scores (*p* < 0.001) and lower GCS scores (*p* < 0.001). Moreover, these patients had lower ASPECTS scores (p < 0.001). Regarding vascular occlusion distribution, the poor outcome group had a significantly higher proportion of ICA occlusions and tandem lesions (*p* = 0.008). Regarding procedural characteristics, patients in the poor outcome group had longer PRT (*p* < 0.001), required more mechanical thrombectomy attempts (*p* = 0.047), and had lower successful reperfusion rates (*p* = 0.006).

### 3.2 Temporal evolution of laboratory parameters

**Figure 1:**
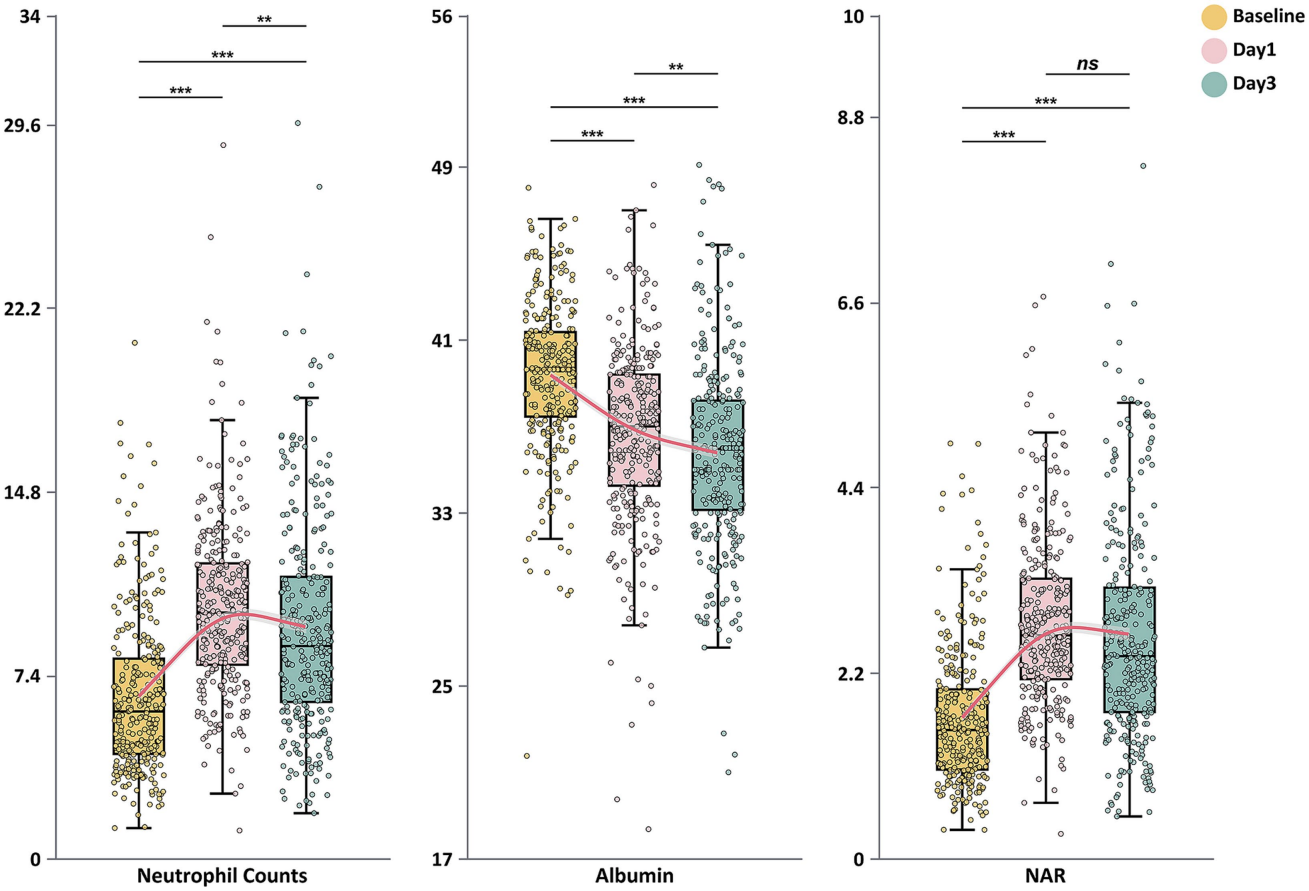
Dynamic changes in neutrophil counts, serum albumin levels, and neutrophil-to-albumin ratio during the first 3 days after endovascular therapy. Box plots show the temporal evolution of neutrophil counts (×10^9^/L), serum albumin (g/L), and neutrophil-to-albumin ratio (NAR) at baseline, day 1, and day 3 after endovascular treatment. The boxes represent the interquartile range (IQR), with the horizontal line indicating the median. Whiskers extend to 1.5 times the IQR, and individual points represent outliers. Red lines connect the median values across time points. Statistical significance: *p < 0.05; **p < 0.01; ***p < 0.001; ns, not significant.

Analysis of the dynamic changes in inflammatory and nutritional markers across the entire study cohort (Figure 1) revealed distinct temporal evolution patterns for neutrophil counts, albumin levels, and NAR. Neutrophil counts increased significantly from baseline to peak values on day 1 (*p* < 0.001), followed by a slight decrease on day 3 (*p* < 0.01). In contrast, albumin concentrations showed a continuous downward trend throughout the observation period, decreasing significantly from baseline to day 1 (*p* < 0.001) and further declining by day 3 (*p* < 0.001). NAR values rose significantly from baseline to day 1 (*p* < 0.001) and maintained significantly higher levels on day 3, with no statistically significant difference between days 1 and 3 (*p* > 0.05).

### 3.3 Association of laboratory parameters with clinical outcomes

Inflammatory biomarkers exhibited significant differences between outcome groups (Table 2). Baseline lymphocyte counts were significantly lower in the poor outcome group compared to the good outcome group (*p* < 0.001). Neutrophil counts were consistently higher in the poor outcome group at all time points (*p* = 0.001), from baseline through day 1 (*p* < 0.001) and day 3 (*p* < 0.001). Both groups reached peak neutrophil counts on postoperative day 1, but the peak was significantly higher in the poor outcome group. By day 3, despite a declining trend in both groups, the poor outcome group maintained significantly higher levels (*p* < 0.001).

**Table 2:** Laboratory parameters according to 90-day functional outcome.

Variables	Overall (*n* = 299)	Good outcome (*n* = 102)	Poor outcome (*n* = 197)	*P*-value
White blood cell, ×10^9^/L	8.3 (6.5, 10.4)	7.9 (6.5, 9.6)	8.6 (6.6, 10.7)	0.058
Lymphocyte, ×10^9^/L	1.50 (1.09, 2.12)	1.76 (1.27, 2.31)	1.40 (0.97, 1.99)	**<0.001**
Red blood cell, ×10^12^/L	4.48 ± 0.67	4.55 ± 0.64	4.44 ± 0.68	0.153
Hemoglobin, g/L	137 (124, 148)	139 (126, 150)	136 (122, 148)	0.203
Platelet count, ×10^9^/L	201 (166, 235)	210 (171, 236)	192 (165, 234)	0.076
Total protein, g/L	71 (68, 75)	71 (68, 74)	71 (68, 75)	0.827
Triglycerides, mmol/L	1.29 (0.87, 1.71)	1.30 (1.02, 1.60)	1.26 (0.81, 1.71)	0.465
Total cholesterol, mmol/L	4.72 (3.94, 5.20)	4.71 (4.00, 5.18)	4.72 (3.89, 5.20)	0.762
HDL cholesterol, mmol/L	1.16 (1.00, 1.31)	1.16 (1.04, 1.30)	1.16 (0.98, 1.32)	0.584
LDL cholesterol, mmol/L	3.09 (2.50, 3.53)	3.09 (2.60, 3.49)	3.09 (2.45, 3.54)	0.586
Creatinine, μmol/L	78 (63, 88)	79 (64, 88)	77 (61, 88)	0.800
Uric acid, μmol/L	407 (334, 452)	414 (332, 452)	403 (335, 449)	0.558
Neutrophil counts, ×10^9^/L				
Baseline	5.9 (4.2, 8.1)	5.1 (3.9, 6.8)	6.4 (4.5, 8.8)	**0.001**
Day 1	9.9 (7.8, 11.9)	8.6 (6.8, 10.8)	10.4 (8.5, 12.5)	**<0.001**
Day 3	8.6 (6.3, 11.4)	6.0 (4.7, 8.1)	9.7 (7.5, 13.3)	**<0.001**
Average	8.3 (6.7, 10.4)	6.8 (5.4, 8.4)	9.07 (7.5, 11.2)	**<0.001**
Albumin, g/L				
Baseline	39.6 (37.5, 41.4)	39.7 (38.1, 40.9)	39.6 (37.1, 41.5)	0.353
Day 1	37.0 (34.3, 39.4)	37.1 (35.2, 39.5)	37.0 (33.5, 39.3)	0.378
Day 3	36.0 (33.1, 38.2)	36.0 (34.6, 38.4)	35.0 (32.5, 38.1)	**0.027**
Average	37.6 (35.4, 39.3)	37.8 (36.3, 39.2)	37.4 (34.9, 39.4)	0.090
Neutrophil-to-albumin ratio				
Baseline	1.53 (1.06, 2.01)	1.32 (0.99, 1.69)	1.64 (1.12, 2.22)	**<0.001**
Day 1	2.65 (2.13, 3.32)	2.34 (1.78, 2.79)	2.86 (2.30, 3.58)	**<0.001**
Day 3	2.40 (1.74, 3.22)	1.66 (1.34, 2.39)	2.78 (2.20, 3.75)	**<0.001**
Average	2.25 (1.78, 2.87)	1.83 (1.44, 2.25)	2.46 (2.03, 3.13)	**<0.001**

Data are presented as median (interquartile range) for continuous variables and number (percentage) for categorical variables. HDL, high-density lipoprotein; LDL, low-density lipoprotein.

Although baseline albumin levels were similar between groups (*p* = 0.353), both groups showed progressive decreases over time. By day 3, the between-group difference became significant, with lower levels in the poor outcome group (*p* = 0.027). NAR displayed distinctive temporal evolution characteristics between outcome groups. The poor outcome group exhibited higher NAR values at all time points, beginning at baseline (*p* < 0.001) and reaching a peak on day 1 (*p* < 0.001). This elevation persisted through day 3 (*p* < 0.001).

### 3.4 Multivariable analysis

Multivariable logistic regression analysis demonstrated that NAR values at all time points were independently associated with 90-day poor functional outcomes (Table 3). The strength of these associations exhibited a temporal gradient, with day 3 NAR and average NAR showing the strongest correlations with outcomes. In the fully adjusted model 3, baseline NAR (OR = 1.85, 95% CI: 1.23–2.78, *p* = 0.003) and day 1 NAR (OR = 1.77, 95% CI: 1.28–2.44, *p* < 0.001) remained independent predictors of poor outcomes. Notably, day 3 NAR (OR = 2.81, 95% CI: 1.96–4.03, *p* < 0.001) and average NAR (OR = 3.49, 95% CI: 2.18–5.58, *p* < 0.001) demonstrated substantially stronger associations with poor outcomes.

**Table 3:** Multivariable logistic regression analysis of NAR at different time points for predicting poor functional outcome.

Variables	Model 1		Model 2		Model 3	
	OR (95% CI)	*P*-value	OR (95% CI)	*P*-value	OR (95% CI)	*P*-value
Baseline NAR	1.78 (1.26–2.51)	<0.001	1.85 (1.25–2.76)	0.002	1.85 (1.23–2.78)	0.003
Day 1 NAR	1.97 (1.46–2.64)	<0.001	1.77 (1.29–2.43)	<0.001	1.77 (1.28–2.44)	<0.001
Day 3 NAR	3.10 (2.23–4.33)	<0.001	2.78 (1.95–3.95)	<0.001	2.81 (1.96–4.03)	<0.001
Average NAR	3.91 (2.55–5.99)	<0.001	3.39 (2.15–5.37)	<0.001	3.49 (2.18–5.58)	<0.001

Model 1: unadjusted analysis. Model 2: adjusted for age, diabetes mellitus, atrial fibrillation, baseline National Institutes of Health Stroke Scale score, Glasgow Coma Scale score, and Alberta Stroke Program Early CT Score. Model 3: further adjusted for occlusion site, puncture-to-reperfusion time, number of thrombectomy attempts, reperfusion status, and lymphocyte count. NAR, neutrophil-to-albumin ratio.

### 3.5 Non-linear association analysis

**Figure 2:**
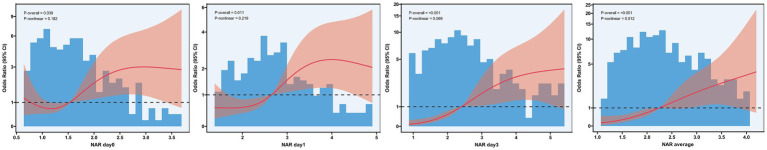
Restricted cubic spline analysis of the association between neutrophil-to-albumin ratio and risk of poor functional outcome after endovascular therapy. Restricted cubic spline regression analysis showing the nonlinear relationships between NAR measured at baseline, day 1, day 3, and the average of all time points (D) with the risk of 90-day poor functional outcome. The solid red line represents the adjusted odds ratio, and the pink shaded area represents the 95% confidence interval. The reference line (odds ratio = 1) is indicated by the dashed horizontal line. Blue histograms show the distribution of NAR values in the study population. P-overall indicates the significance of the overall association, while P-nonlinear indicates the significance of nonlinear components.

To investigate potential non-linear relationships between NAR and 90-day functional outcomes, we conducted RCS analysis (Figure 2). NAR values at all time points demonstrated statistically significant associations with poor functional outcome risk (all P-overall<0.05), though these associations exhibited varying degrees of non-linearity. The non-linear associations for baseline NAR, day 1 NAR, and average NAR did not reach statistical significance (*P*-nonlinear = 0.182, *P*-nonlinear = 0.219, and *P*-nonlinear = 0.512, respectively). Day 3 NAR demonstrated a more pronounced overall association with poor outcomes (*P*-overall<0.001), with its non-linear association approaching the threshold for statistical significance (*P*-nonlinear = 0.069). The curve morphology indicated that as day 3 NAR values increased, the risk of poor outcomes rose at an accelerating rate.

### 3.6 Identification of NAR trajectory patterns

**Figure 3:**
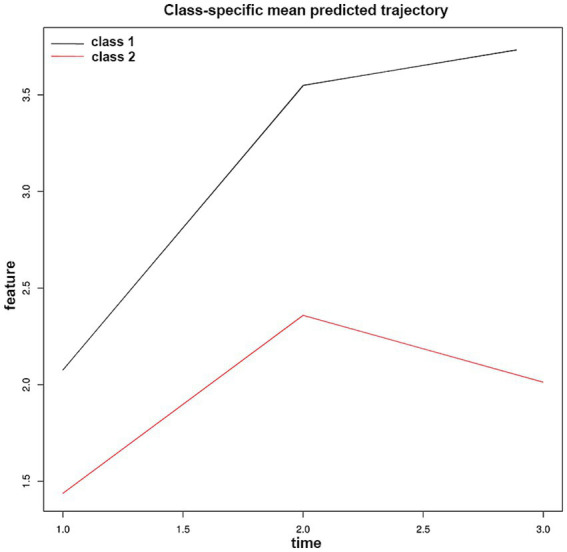
Neutrophil-to-albumin ratio trajectory patterns identified by latent class trajectory modeling in patients with anterior circulation large vessel occlusion stroke after endovascular therapy. Class-specific mean predicted trajectories of neutrophil-to-albumin ratio (NAR) evolution during the first 72 h after endovascular therapy. Two distinct trajectory patterns were identified through latent class trajectory modeling: Class 1 (black line, 31.1% of patients) represents a “continuously increasing” pattern. Class 2 (red line, 68.7% of patients) exhibits a “peak-then-decline” pattern.

To explore the dynamic evolution characteristics of NAR after EVT, we applied LCTM analysis and successfully identified two distinct NAR evolution patterns (Figure 3 and Table 4). Trajectory 1 (31.1%) represented a “continuously increasing” pattern, characterized by relatively high baseline NAR levels (median 1.92) that continued to rise post-procedure, reaching a significant peak on day 3 (median 3.93). In contrast, trajectory 2 (68.7%) exhibited a “peak-then-decline” pattern. Patients in this group had lower baseline NAR levels (median 1.36), reached a moderate peak on day 1 post-procedure (median 2.39), followed by a notable decrease on day 3 (median 2.06).

**Table 4:** Baseline characteristics and laboratory parameters of patients according to NAR trajectory groups before and after propensity score matching.

Variables	Before PSM		*P*-value	After PSM		*P*-value
	Trajectory 1 (*n* = 93)	Trajectory 2 (*n* = 206)		Trajectory 1 (*n* = 63)	Trajectory 2 (*n* = 63)	
Age, years	69 (59, 78)	67(58, 75)	0.327	69 (61, 78)	69 (57, 77)	0.650
Male sex	54 (58.06)	140 (67.96)	0.097	42 (66.67)	42 (66.67)	1.000
Current smoker	26 (27.96)	78 (37.86)	0.096	23 (36.51)	24 (38.10)	0.854
Alcohol consumption	18 (19.35)	53 (25.73)	0.231	13 (20.63)	12 (19.05)	0.823
Medical history						
Hypertension	66 (70.97)	135 (65.53)	0.354	41 (65.08)	39 (61.90)	0.711
Diabetes mellitus	30 (32.26)	54 (26.21)	0.282	20 (31.75)	16 (25.40)	0.430
Hyperlipidemia	17 (18.28)	45 (21.84)	0.481	11 (17.46)	12 (19.05)	0.818
Atrial fibrillation	43 (46.24)	90 (43.69)	0.682	30 (47.62)	31 (49.21)	0.859
Previous stroke/TIA	15 (16.13)	33 (16.02)	0.981	8 (12.70)	12 (19.05)	0.329
Coronary artery disease	17 (18.28)	23 (11.17)	0.094	8 (12.70)	13 (20.63)	0.232
Clinical presentation						
SBP, mmHg	146 (132, 161)	150 (134, 165)	0.203	145 (135, 157)	148 (131, 162)	0.561
DBP, mmHg	88 (77, 96)	87 (77, 97)	0.940	89 (77, 99)	87 (77, 102)	0.984
Baseline NIHSS score	17 (14, 21)	14 (11, 18)	<0.001	16 (13, 20)	17 (13, 19)	0.945
Baseline GCS score	11 (9, 14)	13 (10, 15)	0.003	12 (10, 14)	11 (10, 14)	0.796
Baseline ASPECT score	9 (7, 10)	9.00 (8, 10)	0.476	9 (7, 10)	9.00 (7, 10)	0.974
Stroke etiology			0.964			1.000
LAA	44 (47.31)	94 (45.63)		26 (41.27)	27 (42.86)	
Cardioembolism	45 (48.39)	103 (50.00)		34 (53.97)	33 (52.38)	
Other	4 (4.30)	9 (4.37)		3 (4.76)	3 (4.76)	
Occlusion site			0.631			0.254
ICA	27 (29.03)	48 (23.30)		18 (28.57)	10 (15.87)	
MCA M1	17 (18.28)	37 (17.96)		10 (15.87)	16 (25.40)	
MCA M2	43 (46.24)	101 (49.03)		31 (49.21)	31 (49.21)	
Tandem lesions	27 (29.03)	48 (23.30)		18 (28.57)	10 (15.87)	
Procedural characteristics						
Intravenous thrombolysis	43 (46.24)	86 (41.75)	0.468	27 (42.86)	23 (36.51)	0.466
OPT, min	369 (272, 485)	367 (246, 553)	0.745	340 (259, 556)	385 (227, 538)	0.986
PRT, min	85 (66, 116)	75 (50, 97)	<0.001	80 (63, 104)	80 (51, 100)	0.295
ORT, min	459 (365, 627)	450 (315, 637)	0.408	435 (353, 664)	478 (302, 624)	0.979
NOTA	2 (1, 3)	2 (1, 2)	0.120	2 (1, 3)	2 (1, 3)	0.613
Successful reperfusion	73 (78.49)	182 (88.35)	0.026	53 (84.13)	55 (87.30)	0.611
Treatment strategy			0.296			0.233
Stent retriever	25 (26.88)	43 (20.87)		12 (19.05)	16 (25.40)	
Aspiration	2 (2.15)	14 (6.80)		2 (3.17)	7 (11.11)	
Combined approach	61 (65.59)	136 (66.02)		46 (73.02)	38 (60.32)	
Balloon angioplasty	18 (19.35)	32 (15.53)	0.412	10 (15.87)	8 (12.70)	0.611
Laboratory parameters						
White Blood Cell, ×10^9^/L	10.15 (7.54, 13.12)	7.76 (6.33, 9.39)	<0.001	8.53 (6.93, 10.56)	8.98 (6.97, 10.69)	0.884
Lymphocyte, ×10^9^/L	1.40 (0.88, 1.73)	1.60 (1.14, 2.24)	0.008	1.44 (1.17, 1.73)	1.45 (0.98, 1.99)	0.884
Red Blood Cell, ×10^12^/L	4.28 (3.83, 4.71)	4.55 (4.15, 4.94)	0.001	4.46 (4.02, 4.81)	4.29 (4.06, 4.88)	0.924
Hemoglobin, g/L	133 (116, 143)	139 (126, 150)	0.004	137 (125, 144)	137 (127, 147)	0.930
Platelet count, ×10^9^/L	203 (167, 249)	201 (166, 230)	0.298	197 (169, 240)	203 (163, 252)	0.849
Total Protein, g/L	71.4 (68.6, 74.7)	71.4 (67.9, 74.5)	0.777	71.4 (69.0, 74.1)	71.42 (67.6, 73.5)	0.289
Triglycerides, mmol/L	1.35 (0.86, 1.73)	1.29 (0.88, 1.61)	0.870	1.35 (0.85, 1.86)	1.14 (0.75, 1.52)	0.118
Total Cholesterol, mmol/L	4.72 (3.55, 5.20)	4.72 (3.99, 5.19)	0.480	4.72 (4.00, 5.46)	4.72 (3.84, 5.14)	0.651
HDL cholesterol, mmol/L	1.14 (0.95, 1.29)	1.18 (1.03, 1.33)	0.134	1.16 (0.95, 1.34)	1.20 (1.08, 1.28)	0.459
LDL cholesterol, mmol/L	3.09 (2.23, 3.60)	3.09 (2.60, 3.48)	0.603	3.09 (2.49, 3.62)	3.09 (2.53, 3.34)	0.676
Creatinine, μmol/L	78.4 (58.6, 88.1)	76.7 (63.6, 87.5)	0.787	78.4 (61.2, 84.1)	79.0 (64.5, 90.8)	0.584
Uric acid, μmol/L	407 (338, 463)	407 (332, 448)	0.696	409 (356, 474)	414 (357, 434)	0.543

Values are presented as median (interquartile range) or number (percentage). SPECTS, Alberta Stroke Program Early CT Score; DBP, diastolic blood pressure; GCS, Glasgow Coma Scale; HDL, high-density lipoprotein; ICA, internal carotid artery; LAA, large-artery atherosclerosis; LDL, low-density lipoprotein; MCE, malignant cerebral edema; NAR, neutrophil-to-albumin ratio; NIHSS, National Institutes of Health Stroke Scale; NOTA, number of thrombectomy attempts; OPT, onset-to-puncture time; ORT, onset-to-reperfusion time; PRT, puncture-to-reperfusion time; SBP, systolic blood pressure; TIA, transient ischemic attack.

### 3.7 Propensity score-matched analysis of NAR trajectory groups

After identifying two distinct NAR evolution patterns using LCTM, we compared baseline characteristics between patients in the “continuously increasing” trajectory (trajectory 1, *n* = 93) and the “peak-then-decline” trajectory (trajectory 2, *n* = 206) (Table 4). Pre-matching analysis revealed that trajectory 1 patients had more severe neurological deficits (baseline NIHSS score: 17 vs. 14, *p* < 0.001; GCS score: 11 vs. 13, *p* = 0.003). Regarding procedure-related parameters, trajectory 1 group had significantly longer PRT (85 vs. 75 min, *p* < 0.001) and lower rates of successful reperfusion (78.49% vs. 88.35%, *p* = 0.026). Laboratory indices showed that trajectory 1 patients exhibited more pronounced inflammatory and nutritional imbalance, characterized by significantly elevated baseline white blood cell counts (*p* < 0.001), decreased lymphocyte counts (*p* = 0.008), and reduced red blood cell counts (*p* = 0.001) and hemoglobin levels (*p* = 0.004).

To control for potential selection bias due to baseline characteristic imbalances, we employed PSM for 1:1 exact matching, ultimately including 63 patients in each group. After matching, both groups achieved good balance in demographic characteristics, medical history, clinical presentation, procedure-related parameters, and laboratory indices (all *p* > 0.05). Specifically, baseline NIHSS score (16 vs. 17, *p* = 0.945), GCS score (12 vs. 11, *p* = 0.796), PRT (80 vs. 80 min, *p* = 0.295), successful reperfusion rate (84.13% vs. 87.30%, *p* = 0.611), and various inflammatory and nutritional indicators were effectively balanced.

### 3.8 Clinical outcomes according to NAR trajectories

After PSM, we compared clinical outcomes between the two NAR trajectory groups (Table 5). In the matched cohort, patients exhibiting trajectory 1 (continuously increasing) demonstrated significantly worse outcomes. The trajectory 1 group had a higher incidence of poor 90-day functional outcomes (*p* = 0.008). Similarly, the rates of sICH (*p* = 0.010), MCE (*p* < 0.001), and IHM (*p* < 0.001) were all significantly elevated in the trajectory 1 group.

**Table 5:** Comparison of clinical outcomes between NAR trajectory groups before and after propensity score matching.

Variables	Before PSM		*P*-value	After PSM		*P*-value
	Trajectory 1 (*n* = 93)	Trajectory 2 (*n* = 206)		Trajectory 1 (*n* = 63)	Trajectory 2 (*n* = 63)	
Neutrophil, ×10^9^/L						
Baseline	7.70 (5.57, 11.41)	5.29 (3.93, 7.01)	<0.001	6.38 (4.71, 8.67)	6.97 (4.53, 8.71)	0.884
Day 1	13.12 (11.06, 14.64)	8.77 (6.96, 10.36)	<0.001	12.46 (10.91, 14.79)	8.98 (7.41, 10.35)	<0.001
Day 3	13.89 (10.73, 16.29)	7.24 (5.62, 8.88)	<0.001	13.92 (10.46, 16.71)	7.99 (6.08, 9.69)	<0.001
Average	11.42 (10.75, 12.96)	7.18 (5.99, 8.51)	<0.001	11.31 (10.07, 11.93)	8.08 (6.67, 9.36)	<0.001
Albumin, g/L						
Baseline	39.5 (36.6, 41.2)	39.6 (37.7, 41.4)	0.137	39.3 ± 3.1	39.4 ± 3.5	0.784
Day 1	35.8 (32.8, 38.7)	37.3 (35.1, 39.7)	0.004	37.0 (34.2, 38.9)	37.2 (34.4, 39.0)	0.764
Day 3	33.89 (31.9, 36.0)	36.0 (34.2, 38.9)	<0.001	34.2 (31.9, 36.6)	36.0 (34.7, 38.3)	0.004
Average	36.5 (33.7, 38.5)	37.9 (36.1, 39.5)	<0.001	36.82 (34.59, 38.54)	37.7 (35.8, 39.3)	0.154
NAR						
Baseline	1.92 (1.41, 2.88)	1.36 (0.99, 1.76)	<0.001	1.61 (1.25, 2.21)	1.77 (1.21, 2.23)	0.982
Day 1	3.78 (3.18, 4.34)	2.39 (1.88, 2.79)	<0.001	3.60 (2.96, 4.28)	2.50 (2.04, 2.86)	<0.001
Day 3	3.93 (3.19, 4.94)	2.06 (1.53, 2.50)	<0.001	3.98 (3.16, 4.95)	2.31 (1.73, 2.60)	<0.001
Average	3.23 (2.97, 3.70)	1.96 (1.61, 2.27)	<0.001	3.09 (2.87, 3.45)	2.24 (1.85, 2.44)	<0.001
Clinical outcome						
Poor outcome	81 (87.10)	116 (56.31)	<0.001	53 (84.13)	40 (63.49)	0.008
HT	50 (53.76)	79 (38.35)	0.013	37 (58.73)	30 (47.62)	0.211
sICH	37 (39.78)	31 (15.05)	<0.001	24 (38.10)	11 (17.46)	0.010
MCE	40 (43.01)	28 (13.59)	<0.001	24 (38.10)	6 (9.52)	<0.001
IHM	37 (39.78)	14 (6.80)	<0.001	24 (38.10)	3 (4.76)	<0.001

Values are presented as median (interquartile range) or number (percentage). HT, hemorrhagic transformation; IHM, in-hospital mortality; MCE, malignant cerebral edema; PSM, propensity score matching; sICH, symptomatic intracerebral hemorrhage.

Univariate conditional logistic regression analysis confirmed significant associations between NAR trajectory patterns and clinical outcomes in the PSM cohort (Table 6). Compared to patients with continuously increasing NAR (trajectory 1), those exhibiting an initial increase followed by subsequent decrease (trajectory 2) had significantly reduced risk of poor functional outcomes (OR = 0.38, 95% CI: 0.17–0.86, *p* = 0.020) and sICH (OR = 0.38, 95% CI: 0.17–0.86, *p* = 0.020). This protective association was even more pronounced for MCE (OR = 0.25, 95% CI: 0.10–0.61, *p* = 0.002) and IHM (OR = 0.13, 95% CI: 0.04–0.42, *p* < 0.001).

**Table 6:** Univariate analysis of NAR trajectory patterns and clinical outcomes in the propensity score-matched cohort.

Variables	OR (95% CI)	*P*-value
Poor outcome		0.020
Trajectory 1	1.00 (Reference)	
Trajectory 2	0.38 (0.17–0.86)	
sICH		0.020
Trajectory 1	1.00 (Reference)	
Trajectory 2	0.38 (0.17–0.86)	
MCE		0.002
Trajectory 1	1.00 (Reference)	
Trajectory 2	0.25 (0.10–0.61)	
IHM		<0.001
Trajectory 1	1.00 (Reference)	
Trajectory 2	0.13 (0.04–0.42)	

Results from univariate conditional logistic regression analysis in the propensity score-matched cohort (*n* = 126; 63 patients per trajectory group). Trajectory 1 (reference group) represents a continuously increasing neutrophil-to-albumin ratio (NAR) pattern characterized by relatively high baseline values that continued to rise through day 3 post-procedure. Trajectory 2 represents a peak-then-decline pattern with lower baseline NAR that peaked on day 1 and subsequently decreased on day 3. CI, confidence interval; NAR, neutrophil-to-albumin ratio; OR, odds ratio. HT, hemorrhagic transformation; IHM, in-hospital mortality; MCE, malignant cerebral edema; PSM, propensity score matching; sICH, symptomatic intracerebral hemorrhage.

### 3.9 Predictive performance of NAR for clinical outcomes

**Figure 4:**
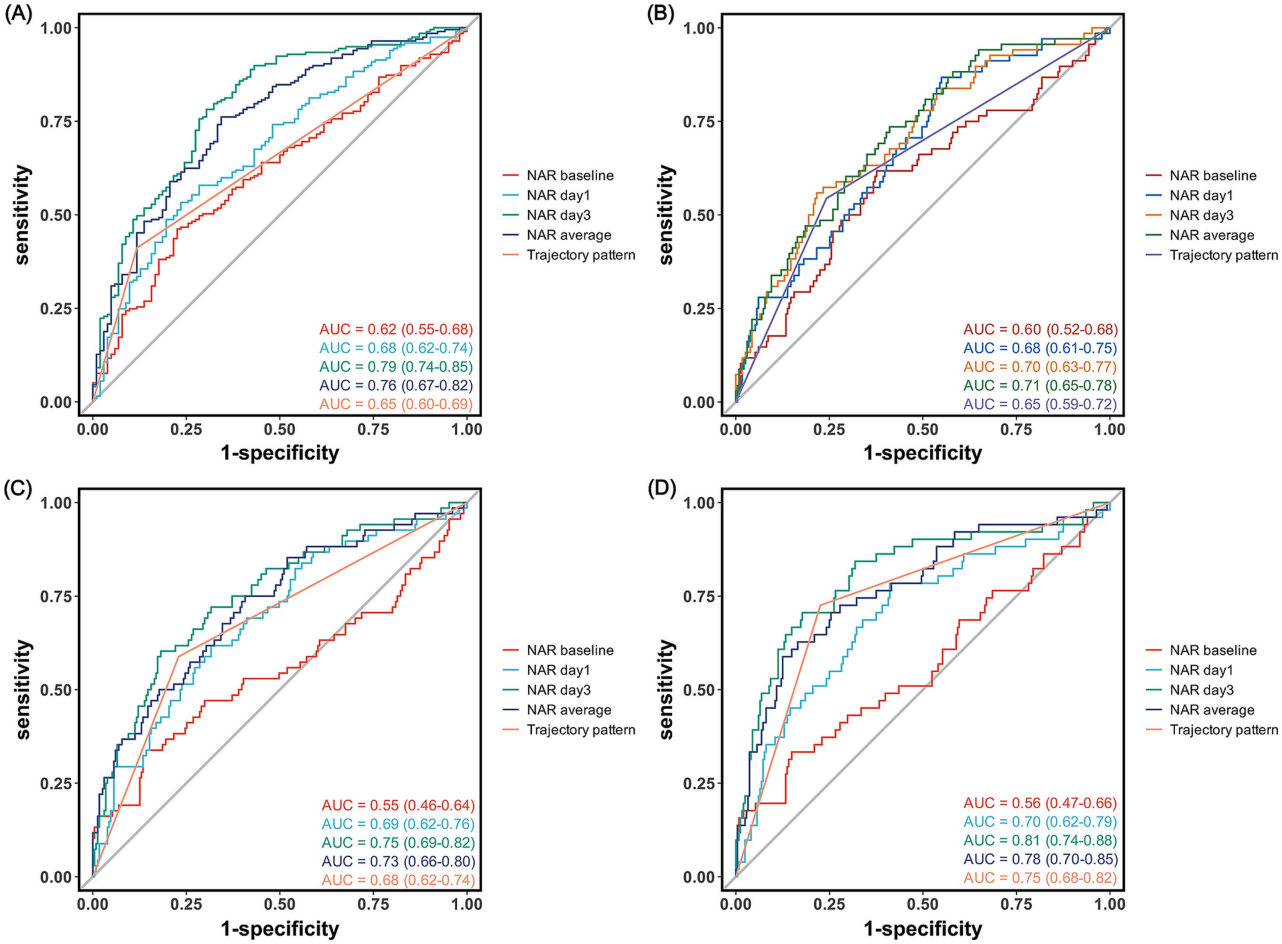
Receiver operating characteristic curves for neutrophil-to-albumin ratio in predicting clinical outcomes after endovascular therapy. Receiver operating characteristic curves showing the predictive performance of NAR at different time points for **(A)** 90-day poor functional outcome (modified Rankin Scale score 3–6), **(B)** symptomatic intracranial hemorrhage, **(C)** malignant cerebral edema, and **(D)** in-hospital mortality.

The predictive value of NAR for clinical outcomes demonstrated significant time-dependent characteristics (Table 7 and Figure 4). For predicting 90-day poor outcomes, the discriminative ability of NAR progressively increased over time: the AUC rose from 0.62 at baseline to 0.68 on day 1, reaching its highest value of 0.79 on day 3. day 3 NAR not only exhibited the best discriminative ability but also achieved high accuracy (0.75), with sensitivity and specificity of 0.70 and 0.78, respectively, at a cutoff value of 2.13. In comparison, the predictive performance based on trajectory pattern classification (AUC = 0.65), though slightly lower than single time-point NAR, provided higher sensitivity (0.88).

**Table 7:** Diagnostic performance of NAR for predicting clinical outcome.

NAR parameter	AUC (95% CI)	Accuracy (95% CI)	Sensitivity (95% CI)	Specificity (95% CI)	Cut-off value
Poor outcome					
Baseline	0.62 (0.55–0.68)	0.57 (0.51–0.63)	0.77 (0.69–0.86)	0.46 (0.39–0.53)	1.72
Day 1	0.68 (0.62–0.74)	0.61 (0.55–0.67)	0.76 (0.68–0.85)	0.53 (0.46–0.60)	2.82
Day 3	0.79 (0.74–0.85)	0.75 (0.70–0.80)	0.70 (0.61–0.79)	0.78 (0.72–0.84)	2.13
Average	0.76 (0.70–0.81)	0.73 (0.67–0.78)	0.66 (0.56–0.75)	0.76 (0.70–0.82)	2.02
Trajectory pattern	0.65 (0.60–0.69)	0.57 (0.51–0.63)	0.88 (0.82–0.94)	0.41 (0.34–0.48)	NA
sICH					
Baseline	0.60 (0.52–0.68)	0.62 (0.56–0.68)	0.62 (0.56–0.69)	0.62 (0.50–0.73)	1.64
Day 1	0.68 (0.61–0.75)	0.55 (0.49–0.60)	0.45 (0.39–0.51)	0.87 (0.79–0.95)	2.44
Day 3	0.70 (0.63–0.77)	0.73 (0.68–0.78)	0.78 (0.73–0.84)	0.56 (0.44–0.68)	3.07
Average	0.71 (0.65–0.78)	0.62 (0.56–0.68)	0.59 (0.53–0.65)	0.74 (0.63–0.84)	2.27
Trajectory pattern	0.65 (0.59–0.72)	0.71 (0.65–0.76)	0.76 (0.70–0.81)	0.54 (0.43–0.66)	NA
MCE					
Baseline	0.55 (0.46–0.64)	0.74 (0.69–0.79)	0.86 (0.81–0.90)	0.34 (0.23–0.45)	2.28
Day 1	0.69 (0.61–0.76)	0.67 (0.61–0.72)	0.68 (0.62–0.74)	0.62 (0.50–0.73)	2.91
Day 3	0.75 (0.68–0.82)	0.77 (0.72–0.82)	0.82 (0.77–0.87)	0.60 (0.49–0.72)	3.13
Average	0.73 (0.66–0.80)	0.63 (0.57–0.68)	0.59 (0.53–0.66)	0.75 (0.65–0.85)	2.27
Trajectory pattern	0.68 (0.61–0.74)	0.73 (0.67–0.78)	0.77 (0.72–0.82)	0.59 (0.47–0.71)	NA
IHM					
Baseline	0.56 (0.47–0.66)	0.76 (0.71–0.81)	0.85 (0.81–0.90)	0.33 (0.20–0.46)	2.36
Day 1	0.70 (0.62–0.79)	0.62 (0.56–0.68)	0.59 (0.53–0.65)	0.78 (0.67–0.90)	2.72
Day 3	0.81 (0.74–0.88)	0.80 (0.75–0.85)	0.82 (0.78–0.87)	0.71 (0.58–0.83)	3.16
Average	0.77 (0.70–0.85)	0.83 (0.78–0.87)	0.88 (0.83–0.92)	0.59 (0.45–0.72)	3.07
Trajectory pattern	0.75 (0.68–0.82)	0.77 (0.71–0.81)	0.77 (0.72–0.83)	0.73 (0.60–0.85)	NA

NAR measurements were obtained at baseline (admission), day 1, and day 3 after endovascular treatment. Average NAR represents the mean value of all three time points. Trajectory pattern refers to the categorical classification determined by latent class trajectory modeling. NA indicates not applicable for categorical variables. AUC, area under the receiver operating characteristic curve; CI, confidence interval; NAR, neutrophil-to-albumin ratio.

For secondary outcomes, NAR also demonstrated varying degrees of predictive capability. For predicting IHM, day 3 NAR showed the best performance with an AUC of 0.81, outperforming average NAR (AUC = 0.77) and trajectory pattern (AUC = 0.75). At a cutoff value of 3.16, day 3 NAR predicted IHM with an accuracy of 0.80, with sensitivity and specificity of 0.82 and 0.71, respectively.

For predicting MCE, day 3 NAR similarly demonstrated the best predictive performance (AUC = 0.75), slightly higher than mean NAR (AUC = 0.73). For sICH, NAR generally showed moderate discriminative ability, with average NAR (AUC = 0.71) and day 3 NAR (AUC = 0.70) performing comparably and optimally, with cutoff values of 2.27 and 3.07, respectively.

## 4 Discussion

In this retrospective cohort study of anterior circulation LVO-AIS patients undergoing EVT treatment, we confirmed that dynamic changes in NAR can effectively predict adverse clinical outcomes. Our research findings deepen the understanding of the relationship between the inflammatory-nutritional status and clinical outcomes in this patient population from four aspects. First, we observed that patients exhibited distinctive temporal evolution patterns of neutrophil counts and albumin levels within 3 days after EVT. Neutrophil counts peaked 24 h post-procedure and gradually declined, while albumin levels continuously decreased throughout the entire observation period. Second, the predictive value of NAR for adverse clinical outcomes progressively increased over time, with day 3 measurements showing the strongest predictive ability. Third, the predictive value of NAR remained robust after comprehensive adjustment for confounding factors. Fourth, through LCTM analysis, we identified two distinct NAR evolution patterns: continuously increasing and peak-then-decline, with the continuously increasing pattern significantly associated with adverse clinical outcomes.

Our findings resonate with previous research while expanding the current understanding. In recent years, composite biomarkers integrating inflammatory status and nutritional levels have shown unique advantages in AIS prognosis assessment, with C-reactive protein/albumin ratio and hemoglobin-albumin-lymphocyte-platelet score demonstrating significant predictive value (Akpınar et al., 2023; Chen et al., 2024). As a novel integrated indicator, NAR provides a new perspective for prognostic evaluation by combining neutrophil count with albumin level. Currently, NAR has demonstrated excellent prognostic assessment capability in various disease states, including malignant tumors, acute myocardial infarction, heart failure, hemorrhagic stroke, and autoimmune diseases (Deng et al., 2024; Hu et al., 2022; Kamal et al., 2023; Karasu et al., 2023; Xie et al., 2024). In the field of ischemic stroke, recent studies have preliminarily revealed significant associations between NAR and adverse outcomes. Zhao et al. showed that higher baseline NAR levels were significantly associated with 30-day post-stroke mortality risk (HR = 1.18, 95% CI: 1.07–1.32), while Bao et al. found that baseline NAR had independent value in predicting 90-day functional outcomes (OR = 9.34, 95% CI: 1.09–80.13) (Bao et al., 2024; Zhao et al., 2023).

However, these studies were primarily limited to single time-point NAR measurements, failing to fully capture the dynamic balance of inflammatory-nutritional status. Growing evidence suggests that compared to static measurements, dynamic patterns of inflammatory marker changes more accurately reflect disease progression and clinical outcomes in AIS patients (Gao et al., 2024; Guo et al., 2016; Qian et al., 2024; Weng et al., 2021; Wu and Chen, 2023; Ying et al., 2020; Zhang et al., 2024). Previous studies have found that in patients receiving intravenous thrombolytic therapy, inflammatory marker levels measured 24 h after admission were more closely associated with multiple adverse clinical outcomes compared to baseline measurements, including early neurological deterioration, HT, sICH, 90-day poor functional prognosis, and all-cause mortality (Wu and Chen, 2023; Guo et al., 2016; Zhang et al., 2024). Subsequent studies extended the monitoring time window and demonstrated that the neutrophil-to-lymphocyte ratio measured on day 3 had a significantly better predictive ability for 90-day functional prognosis than baseline or day 1 measurements (Qian et al., 2024). Although day 7 measurements still held prognostic significance, their predictive efficacy was markedly reduced compared to day 1 or day 3 (Ying et al., 2020; Qian et al., 2024; Weng et al., 2021). This time-dependent pattern was further validated in LVO-AIS patients undergoing EVT, where day 3 inflammatory markers exhibited significant predictive value for multiple adverse outcomes, including post-procedural HT, MCE, IHM, and 90-day poor functional status (Gao et al., 2024). Our study, by introducing latent class trajectory modeling analysis, systematically characterized the temporal evolution features and trajectory patterns of NAR after EVT for anterior circulation LVO-AIS for the first time, providing a novel perspective for understanding the complex relationship between inflammatory-nutritional status and clinical outcomes.

Pathophysiological processes in AIS involve a series of complex cascading reactions, including the release of toxic mediators, intracellular calcium overload, free radical generation, neuroinflammation, and neuronal apoptosis (Kuriakose and Xiao, 2020). Among these, neuroinflammation plays a crucial role in both the acute and chronic stages of stroke progression (Cheng et al., 2023; Alsbrook et al., 2023). The inflammatory cascade in AIS patients typically initiates within hours of onset, peaks at 24–48 h, and gradually subsides after 72 h, a temporal pattern that closely aligns with the dynamic changes in neutrophil counts observed in our study (Alsbrook et al., 2023; Tu et al., 2010). Although rapid restoration of cerebral blood flow is the most effective strategy to mitigate ischemic injury, the inflammatory response may aberrantly lead to secondary reperfusion injury. Therefore, for patients undergoing EVT, neutrophil-derived indicators hold special value in risk stratification assessments.

Existing evidence suggests that neutrophils contribute to the pathological processes of stroke through multiple mechanisms, influencing BBB integrity, neuronal injury, cellular apoptosis, and neuroplasticity. As the earliest peripheral immune cells to infiltrate ischemic brain tissue, neutrophils exhibit remarkable functional plasticity and can differentiate into neurotoxic N1 phenotypes or neuroprotective N2 phenotypes (Xie et al., 2023). The N1 phenotype undergoes morphological changes by upregulating the expression of adhesion molecules, thereby achieving transendothelial migration. These cells subsequently engage in directed migration toward the ischemic region under the guidance of chemokine gradients, releasing pro-inflammatory mediators, reactive oxygen species, proteases, and matrix metalloproteinases, ultimately triggering secondary tissue damage (Franck et al., 2018). Furthermore, activated neutrophils release neutrophil extracellular traps, which promote thrombosis and exacerbate BBB dysfunction through multiple mechanisms: enhancing immune cell infiltration, inhibiting angiogenesis and vascular repair, promoting neuronal death, and hindering functional recovery (Kang et al., 2020). Experimental studies have demonstrated that blocking neutrophil infiltration can effectively alleviate secondary brain injury and improve functional prognosis in middle cerebral artery occlusion models (Jickling et al., 2015). Recent targeted therapy studies focusing on neutrophil adhesion and trafficking have shown promising prospects, achieving preliminary success in reducing neutrophil recruitment to brain tissue. However, the effectiveness of these therapeutic strategies still requires further validation (Herz et al., 2015).

Albumin is not only a marker for assessing nutritional status but also a crucial antioxidant under oxidative stress and inflammatory conditions. Through its antioxidant, anti-inflammatory, and antithrombotic properties, albumin plays a key protective role in resisting cerebral infarction (Don and Kaysen, 2004). Albumin can inhibit endothelial cell apoptosis and regulate microvascular permeability, thereby maintaining cerebrovascular integrity and BBB stability (Belayev et al., 2002). However, in critical illness states, processes such as albumin’s capillary leakage rate and its synthesis-degradation balance are significantly affected, resulting in a rapid decline in serum albumin concentrations during the acute phase, followed by a gradual recovery during the disease remission period (Aguirre Puig et al., 2014). Our research findings are highly consistent with these established patterns, with albumin levels showing a persistent downward trend throughout the observation period. Furthermore, a complex bidirectional interaction exists between hypoalbuminemia and the inflammatory response. Decreased albumin levels may trigger inflammatory cascades and leukocytosis, while severe inflammatory reactions suppress hepatic albumin synthesis. This vicious cycle may constitute one of the important mechanisms underlying poor prognosis (Artigas et al., 2016; Sheinenzon et al., 2021).

The stronger predictive value of day 3 NAR compared to baseline and day 1 likely reflects the dynamic evolution characteristics of post-stroke inflammatory responses and stress states. Baseline NAR primarily reflects the initial inflammatory state and may be influenced by factors such as the patient’s pre-onset condition, time of symptom discovery, and comorbidities, limiting its predictive ability. Although day 1 NAR can capture changes in the early inflammatory response, these changes may represent an immediate stress response to ischemic injury rather than accurately reflecting the disease progression trajectory. In contrast, day 3 NAR seems to better characterize early treatment responses and disease progression patterns. At this time point, the acute inflammatory response has fully unfolded, with neutrophil recruitment and infiltration in the ischemic region reaching a peak, accompanied by the manifestation of BBB disruption and secondary injury. Particularly for patients undergoing EVT, day 3 NAR may more comprehensively reflect the extent of reperfusion injury and the persistent, intense inflammatory response. Moreover, day 3 albumin levels not only indicate the intensity of the acute stress response but may also predict the potential for tissue repair and functional reconstruction. Thus, NAR at this time point integrates dynamic change information from both inflammatory and nutritional statuses, providing a more reliable time window for assessing disease severity and predicting prognosis.

Our study identified two distinct NAR dynamic evolution patterns that may reflect different pathophysiological response types following ischemia–reperfusion. The continuously increasing trajectory (trajectory 1) was associated with significantly increased risk of adverse clinical outcomes. This pattern, characterized by relatively high baseline NAR levels that continued to rise through day 3, likely reflects a persistent and worsening inflammatory-nutritional imbalance. In contrast, the peak-then-decline trajectory (trajectory 2) featured lower baseline NAR, which reached a moderate peak on day 1 before subsequently decreasing, potentially representing a controlled, self-limiting inflammatory response. Notably, although trajectory pattern classification showed relatively lower AUC values in ROC analysis, it demonstrated significant clinical predictive value in univariate conditional logistic regression analysis, particularly for IHM and MCE. This suggests that NAR trajectory patterns, as a predictive tool integrating dynamic change information, can provide clinical value not available from single time-point measurements.

Several limitations of this study warrant consideration. First, as a single-center retrospective study, selection and information biases were inevitable. Second, we could not fully control all clinical variables that might influence NAR dynamics. Medications administered during treatment may significantly alter neutrophil counts and their dynamic characteristics; similarly, fluid management strategies and pre-existing nutritional status could substantially impact albumin levels—factors that were not comprehensively assessed or adjusted for in this study. Third, although this study confirmed the predictive value of NAR and its dynamic trajectories for clinical outcomes, we have not directly compared it with other established inflammation-related biomarkers (such as neutrophil-to-lymphocyte ratio and systemic immune-inflammation index). The incremental predictive value of NAR beyond existing prognostic assessment tools remains to be further elucidated. Additionally, this study used telephone follow-up to assess 90-day functional outcomes, which may limit the accuracy of functional status evaluation. Face-to-face assessments might provide more objective and comprehensive functional outcome data. Based on these limitations, future research should design prospective multicenter cohort studies to validate our findings, compare the predictive performance of NAR with other inflammatory markers, and explore individualized intervention strategies based on NAR dynamic monitoring to provide more precise guidance for clinical decision-making.

## 5 Conclusion

In this study, through systematic observation of anterior circulation LVO-AIS patients undergoing EVT, we confirmed that dynamic changes in NAR have independent predictive value for clinical outcomes. Day 3 NAR measurements demonstrated optimal predictive capability, while the two distinct NAR evolution patterns (“continuously increasing” versus “peak-then-decline”) identified by LCTM provided a new perspective for early risk stratification. These findings reveal the crucial role of dynamic inflammatory-nutritional status balance in stroke prognosis, suggesting that dynamic monitoring of NAR can serve as an effective tool for assessing post-EVT patient risk, providing scientific basis for developing precision intervention strategies.

## Data Availability

The datasets presented in this article are not readily available because the datasets used and/or analyzed during the current study are available from the corresponding author upon reasonable request. Requests to access the datasets should be directed to Renjing Zhu, zhurenjing@163.com.
