# Supplementary material for: Distinct trajectory patterns of neutrophil-to-albumin ratio predict clinical outcomes after endovascular therapy in large vessel occlusion stroke
Source: Front Aging Neurosci. 2025 Jun 4;17:1570662. doi: 10.3389/fnagi.2025.1570662 (PMC12174067; doi:10.3389/fnagi.2025.1570662)
Supplement: Supplementary file 1 [file Table_1.DOCX]

| **Supplementary Table 1.** Multicollinearity Assessment of Variables in Multivariable Logistic Regression Models for Clinical Outcomes | | | | | | | | |
| --- | --- | --- | --- | --- | --- | --- | --- | --- |
| **Variable** | **Tolerance^*^** | **VIF^*^** | **Tolerance^**^** | **VIF^**^** | **Tolerance^***^** | **VIF^***^** | **Tolerance^****^** | **VIF^****^** |
| NAR |  |  |  |  |  |  |  |  |
| Day 0 | 0.844 | 1.186 | - | - | - | - | - | - |
| Day 1 | - | - | 0.944 | 1.059 | - | - | - | - |
| Day 3 | - | - | - | - | 0.973 | 1.027 | - | - |
| Average | - | - | - | - | - | - | 0.938 | 1.066 |
| Age | 0.774 | 1.291 | 0.783 | 1.277 | 0.785 | 1.273 | 0.771 | 1.296 |
| Diabetes mellitus | 0.946 | 1.056 | 0.943 | 1.060 | 0.951 | 1.051 | 0.944 | 1.060 |
| Atrial fibrillation | 0.718 | 1.392 | 0.730 | 1.371 | 0.733 | 1.364 | 0.729 | 1.371 |
| NIHSS score | 0.621 | 1.611 | 0.629 | 1.589 | 0.638 | 1.567 | 0.623 | 1.605 |
| GCS score | 0.646 | 1.549 | 0.650 | 1.539 | 0.648 | 1.544 | 0.647 | 1.547 |
| ASPECT score | 0.958 | 1.043 | 0.953 | 1.050 | 0.953 | 1.049 | 0.949 | 1.053 |
| Occlusion site | 0.831 | 1.203 | 0.836 | 1.196 | 0.822 | 1.217 | 0.821 | 1.219 |
| PRT | 0.696 | 1.436 | 0.685 | 1.460 | 0.695 | 1.439 | 0.681 | 1.467 |
| NOTA | 0.790 | 1.266 | 0.783 | 1.277 | 0.816 | 1.225 | 0.797 | 1.255 |
| Successful reperfusion | 0.944 | 1.059 | 0.920 | 1.087 | 0.921 | 1.086 | 0.925 | 1.081 |
| Lymphocyte | 0.902 | 1.108 | 0.958 | 1.044 | 0.957 | 1.045 | 0.952 | 1.050 |
| Variables were assessed for multicollinearity before inclusion in multivariable logistic regression models. Four separate models were constructed, each including one neutrophil-to-albumin ratio (NAR) measurement: baseline (Day 0), Day 1, Day 3, or the average across all three time points. Variance inflation factor (VIF) values <10 and tolerance values >0.1 indicate acceptable multicollinearity levels. All variables met these criteria and were retained in the final models. **Abbreviations:** ASPECTS, Alberta Stroke Program Early CT Score; GCS, Glasgow Coma Scale; NAR, neutrophil-to-albumin ratio; NIHSS, National Institutes of Health Stroke Scale; NOTA, number of thrombectomy attempts; PRT, puncture-to-reperfusion time; VIF, variance inflation factor. | | | | | | | | |
