# Supplementary material for: Distinct trajectory patterns of neutrophil-to-albumin ratio predict clinical outcomes after endovascular therapy in large vessel occlusion stroke
Source: Front Aging Neurosci. 2025 Jun 4;17:1570662. doi: 10.3389/fnagi.2025.1570662 (PMC12174067; doi:10.3389/fnagi.2025.1570662)
Supplement: Supplementary file 2 [file Table_2.DOCX]

| **Supplementary Table 2.** Model Selection Criteria for Latent Class Trajectory Modeling of Serial Neutrophil-to-Albumin Ratio Measurements | | | | | | | |
| --- | --- | --- | --- | --- | --- | --- | --- |
| **Model** | **BIC** | **Log Likelihood** | **Class Distribution (%)** | | | | |
|  |  |  | **Class 1** | **Class 2** | **Class 3** | **Class 4** | **Class 5** |
| Model 1 | 2395.97 | -1166.64 | 31.10 | 68.90 |  |  |  |
| Model 2 | 2401.38 | -1157.94 | 28.09 | 27.09 | 44.82 |  |  |
| Model 3 | 2418.67 | -1155.18 | 46.15 | 24.08 | 20.07 | 9.70 |  |
| Model 4 | 2419.27 | -1144.08 | 3.68 | 14.38 | 20.40 | 45.82 | 15.72 |
| Model selection was based on Bayesian Information Criterion (BIC), log likelihood values, and ensuring each trajectory group contained at least 5% of the study population. Lower BIC values and higher log likelihood values indicate better model fit. Based on these criteria, the two-class model (Model 1) was selected as the optimal model, as it had the lowest BIC value while maintaining adequate representation in each trajectory group. **Abbreviations:** BIC, Bayesian Information Criterion. | | | | | | | |
